# Ecological and Behavioral Implications of Multiple Paternity in the Smooth‐Fronted Caiman in French Guiana

**DOI:** 10.1002/ece3.71337

**Published:** 2025-04-23

**Authors:** Carolin Dittrich, Rosanna Mangione, Olivier Marquis, Eva Ringler, Jérémy Lemaire

**Affiliations:** ^1^ Konrad Lorenz Institute of Ethology, Department of Interdisciplinary Life Science University of Veterinary Medicine Vienna Vienna Austria; ^2^ Department of Behavioral and Cognitive Biology University of Vienna Vienna Austria; ^3^ Division of Behavioural Ecology Institute of Ecology and Evolution, University of Bern Bern Switzerland; ^4^ Centre D'etudes Biologiques de Chizé (CEBC) UMR 7372 CNRS‐La Rochelle Université Villiers en Bois France; ^5^ Muséum National D'histoire Naturelle Parc Zoologique de Paris Paris France

**Keywords:** dwarf caiman, environmental constraints, neotropics, nests, polyandry, resources

## Abstract

The identification of genetic mating systems in a variety of species has challenged the previous view on animal mating patterns over the past decade, resulting in the identification of multiple paternity across all vertebrate classes. In crocodylians, all species that have been investigated demonstrate multiple paternity, which may represent the ancestral state of the clade. The smooth‐fronted caiman, *Paleosuchus trigonatus* , is one of the last species whose genetic mating system has yet to be investigated. In this study, we analyzed genetic samples of the smooth‐fronted caiman in French Guiana, a secretive species that is difficult to observe in the wild. Scute samples were taken from three populations and five groups of neonates that hatched shortly before. Microsatellite markers were used to infer the minimum number of fathers that sired each clutch. Our results clearly show that multiple paternity was common, with 60% of the sampled group of neonates showing a minimum of two sires. The potential ecological and behavioral implications of this finding are discussed, as well as recommendations for future research avenues to elucidate this cryptic species' mating behavior and environmental constraints.

## Graphical abstract

The smooth‐fronted caiman, *Paleosuchus trigonatus* , is one of the last species whose genetic mating system has yet to be investigated. In this study, we analyzed genetic samples of the smooth‐fronted caiman in French Guiana, a secretive species that is difficult to observe in the wild. Although we provide evidence of multiple paternity in our study, this might not be sufficient to maintain genetic diversity, necessitating the monitoring of populations to detect trends and planning conservation interventions.

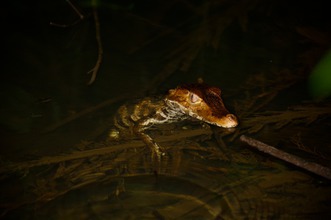

## 1 Introduction

Mating systems exhibit considerable diversity, with the primary distinguishing characteristics being the number of partners an individual engages with and the temporal pattern of mating, which can occur sequentially over a season or once at a specific time point in the reproductive cycle (Kokko et al. 2014). Polygamous matings can be polygynous, which is an association of one male with multiple females, or polyandrous, which is an association of one female with multiple males (Emlen and Oring 1977). Polyandry is very common in many species of both invertebrate and vertebrate animals and often leads to multiple paternity (Pizzari and Wedell 2013; Taylor et al. 2014), where offspring by one female are sired by more than one father. Multiple paternity is found across all vertebrate classes and shows a positive relationship with clutch/litter size (Correia et al. 2021; Dobson et al. 2024). Additionally, it has been confirmed in several reptilian orders, including turtles, squamates, and crocodiles (Isberg 2021; Uller and Olsson 2008). However, whether this reproductive strategy has an evolutionary adaptive value, and the question of the proximate mechanisms involved, remain mostly unanswered (Griffith et al. 2002; Isberg 2021; Taylor et al. 2014; Uller and Olsson 2008).

Some major benefits of multiple paternity for females are an increase in genetic diversity in the offspring, potentially allowing for better adaptability to environmental changes (Jennions and Petrie 2000; Taylor et al. 2014), and thus increasing survival in offspring and increasing female fitness over time. Another common benefit of female multiple matings is increased reproductive success if more (diverse) sperm can fertilize more eggs (Jennions and Petrie 2000), which would also allow post‐copulatory cryptic female choice or sperm competition (Eberhard 2009; Parker 1970). Sperm storage becomes increasingly important in species with low mating encounter rates since sperm limitation could affect female reproduction (Uller and Olsson 2008).

In species where males show strong territoriality and dominance over access to females, the chances for mate choice might be reduced as sexual coercion and sole access to females by the dominant male can be frequently observed (Uller and Olsson 2008). Sexual conflict arises when females and males of a species have different optimal reproductive strategies and costs for mating (Parker 2006). Additionally, environmental and social constraints will affect mating systems and the potential for polygamous mating, depending on resources such as the availability of mates or nest sites and the monopolization of one sex by the other (Emlen and Oring 1977; West‐Eberhard 1983). Whether multiple paternity arises from females choosing different mates based on preferences and adaptive value, or if mate availability determines the rate of multiple paternity without any preference, or if multiple mating is forced on females by dominant males, is not known in most cases, particularly not in secretive species where behavioral observations are scarce or non‐existent.

Until recently, the mating system of most crocodylians was thought to be polygynous, with a single dominant male breeding with several females across his territory (Lang 1987), although the author even states that “In large aggregations, females move with impunity between territories and may court and mate with several dominant males in succession.” With the development of genetic technologies to infer genealogy and the decrease in laboratory costs, genetic mating systems can be inferred in a variety of species without the need for direct behavioral observations. Nowadays, we know that almost all the Old World and New World crocodylians that have been investigated show multiple paternity linked to polyandrous mating (Isberg 2021), which might be the ancestral state of crocodylian mating (Muniz et al. 2019). Nevertheless, crocodylians' mating systems seem to be complex and influenced by environmental and social constraints.

The Smooth‐fronted caiman, *Paleosuchus trigonatus* , is a small‐bodied caiman classified as “least concern” by the IUCN (Campos et al. 2019, Figure 1). Notwithstanding its extensive distribution across the entire Amazon basin, it is one of the least studied of the 26 species of described crocodylians. *P. trigonatus* is primarily a sedentary species inhabiting forest streams under a relatively dense canopy. The species exhibits secretive behavior, with most of its activity occurring during nocturnal hours. During the day, it has been observed to seek refuge in burrows or beneath dead vegetation (Lemaire et al. 2018; Magnusson and Lima 1991). Additionally, the species spends a larger proportion of its time in terrestrial habitat, as shown by a higher amount of terrestrial food items (Magnusson et al. 1987). Consequently, there is a paucity of knowledge regarding their behavior and life history, which may result in an inadequate evaluation of population dynamics and conservation status.

**FIGURE 1:**
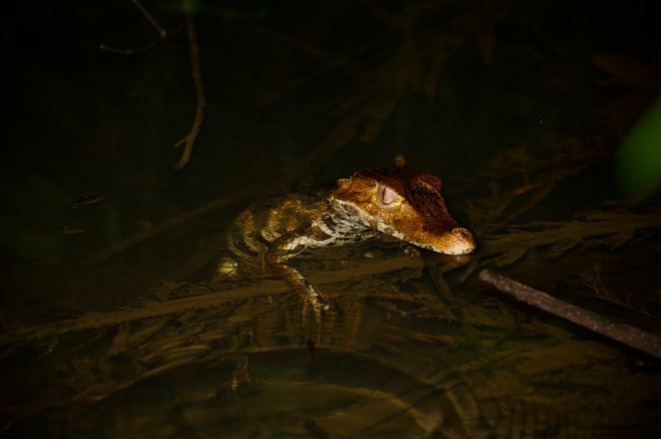
Smooth‐fronted caiman ( *Paleosuchus trigonatus* ) in French Guiana. Credit: Jérémy Lemaire.

Males display both spatial and temporal territoriality, while female home ranges often overlap, indicating a degree of tolerance towards neighboring females (Magnusson and Lima 1991; Marioni et al. 2022). The dominance behavior of males, leading to larger males having increased access to females, might lead to high reproductive skew among males.

Additionally, females traversing male territories with varying probabilities could result in differences in encounter rates for both sexes (Magnusson and Lima 1991). The average number of eggs in a nest is approximately 15 (Magnusson et al. 1985; Thorbjarnarson 1996), and might be positively correlated with female body size (Campos et al. 2015). Females provide parental care by defending their eggs during nighttime against predation during the lengthy incubation period of 100 days (Campos et al. 2016). The post‐hatching parental care is relatively brief compared to other crocodylian species, lasting only 21 days (Magnusson and Lima 1991). The mating system of the species remains unknown; however, it is anticipated to align with polygamy, which seems to be the ancestral state of crocodylians (Muniz et al. 2019).

Here we investigate the genetic mating system of the smooth‐fronted caiman, *P. trigonatus* , in French Guiana. Due to its sedentary and territorial behavior, we expect a low frequency of multiple paternity in this species.

## 2 Methods

### 2.1 Sample Collection

**FIGURE 2:**
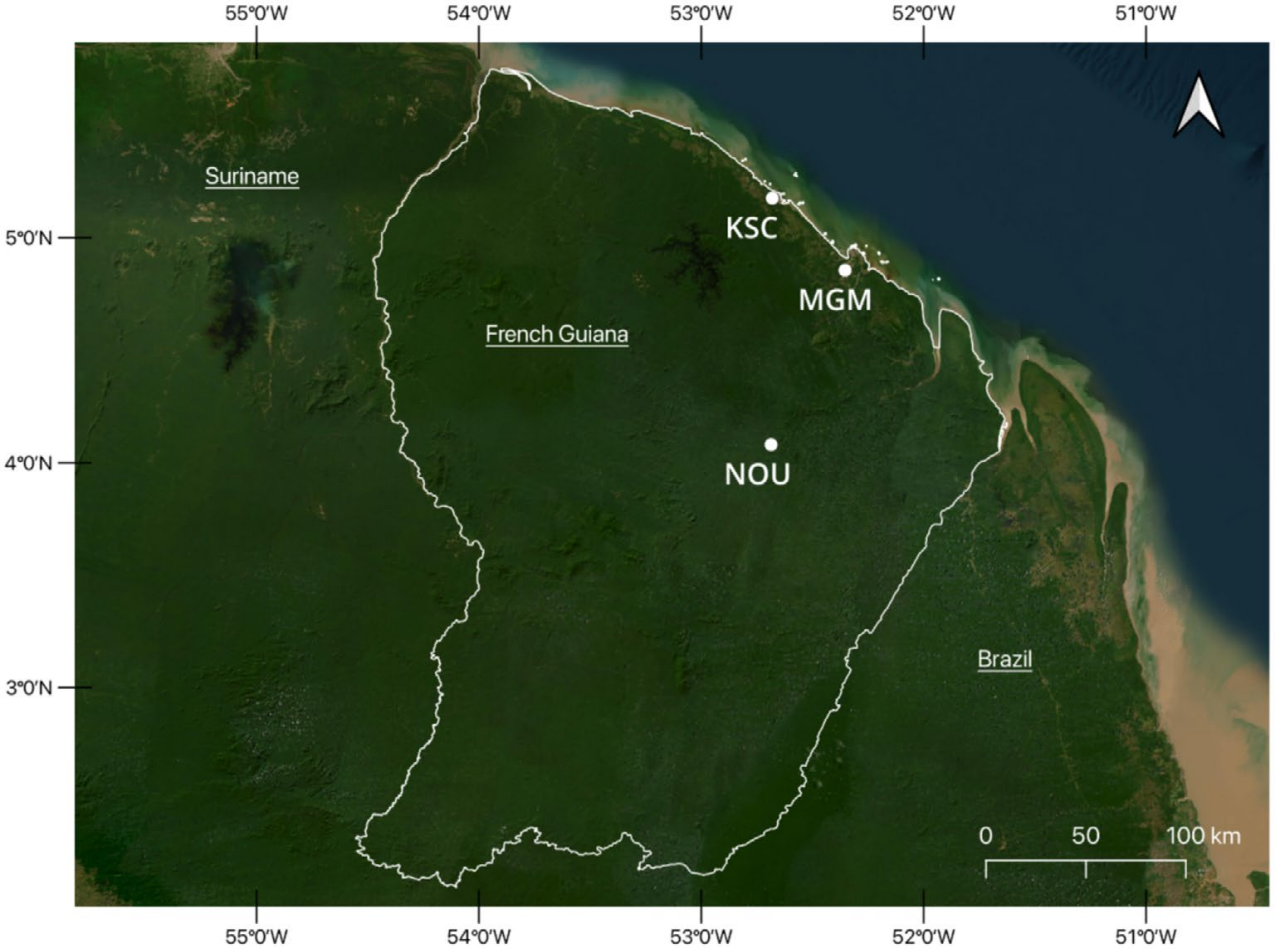
Geographic location of the nests of *Paleosuchus trigonatus* in French Guiana. KSC: Kourou Space Center; MGM: Nature Reserve Mont Grand Matoury; NOU: Nature Reserve Les Nouragues Inselberg. Source map: ESRI Satellite.

**FIGURE 3:**
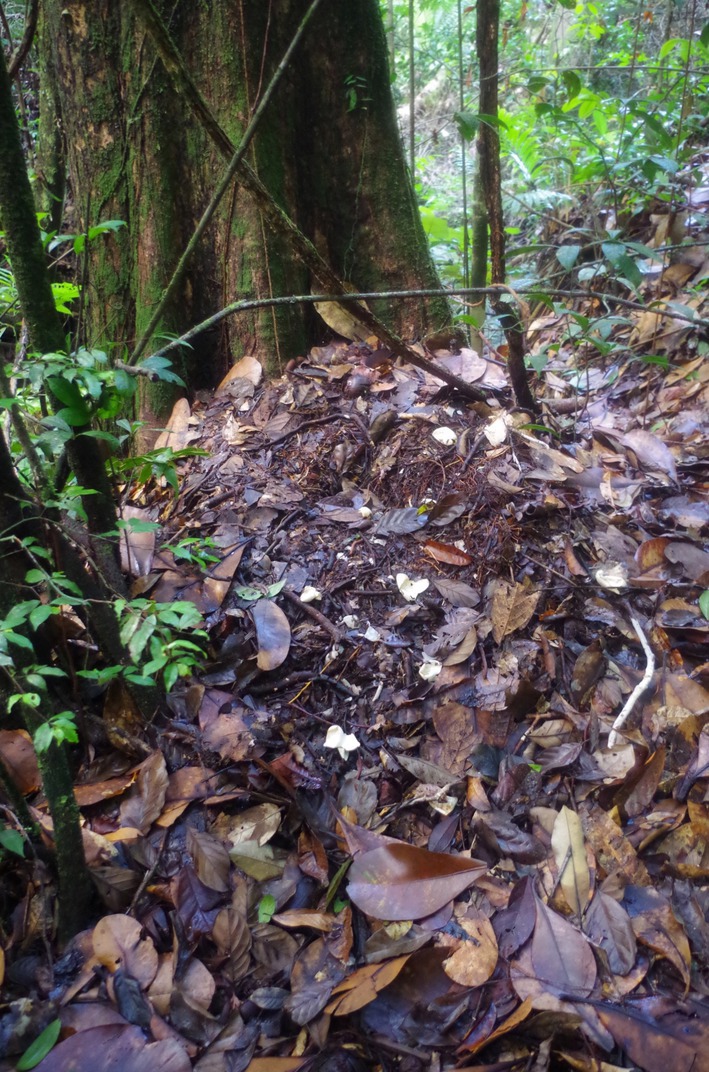
Nest (NOU‐1) and eggshell remains from the smooth‐fronted caiman found in the Les Nouragues nature reserve in 2017. Credit: Jérémy Lemaire.

From April 2016 to February 2020, we identified five freshly hatched nests of *P. trigonatus* in three locations in French Guiana (Figure 2) within the framework of our long‐term caiman monitoring. Neonate caimans were found in small groups within close proximity of each nest. One nest location was found to be reused in two different years (2017 and 2019) at “Les Nouragues – Inselberg” nature reserve. Considering the behavior of young *P. trigonatus* remaining near the nest for up to 21 days with some protection from the mother (Magnusson and Lima 1991), we have no doubt that the young that were found came from the nest located nearby. Furthermore, the remnants of the recently hatched eggs were found at the nest site (Figure 3).

All animals were captured by noose or hand and released at their place of capture immediately after sampling. Tissue samples were taken from tail scutes using clean pliers and were directly preserved in 99% ethanol and further stored at 4°C until DNA extraction. The location of sampled scutes varied, as clipping additionally serves for individual identification, following a marking code that was also applied in our sampling. Nevertheless, the sample taken is always less than 1 cm of the total scute, as described in Lemaire et al. (2021a).

### 2.2 Microsatellite Genotyping

Eight microsatellite loci were chosen following the species‐specific microsatellites developed by Muniz et al. (2019). All details on microsatellites (sequences, repeat motifs, etc.) can be found in the repository. DNA extraction was implemented from preserved scutes by a standard phenol‐chloroform procedure after proteinase K digestion (Sambrook et al. 1989). Polymerase chain reaction (PCR) amplifications were performed on all samples, using reaction volumes of 10 μL containing approximately 10 ng of genomic DNA, 0.2 mmol of each dNTP, 1 μmol of each forward and reverse primer, 0.5 U of Taq DNA polymerase (Axon), and 1 μL of 10× NH4 reaction buffer (Axon), at a final concentration of 1.5 mmol MgCl2. We used the following PCR program: denaturation for 5 min at 95°C, 38 amplification cycles with a denaturation step at 95°C for 45 s, the primer‐specific annealing temperature (58°C for Ptrig8, for all other primers 60°C) for 45 s, 72°C annealing temperature for 45 s, followed by a final annealing extension step for 5 min at 72°C. Differences in the sizes of the amplified alleles and in the fluorescent dye labels of the primers allowed for pooling of multiple loci for the subsequent sequencing process. The pooled products were diluted with water 1:30, mixed with HiDiformamid and the internal size standard ROX500 (Applied Biosystems), and run on an ABI 3130xl Genetic Analyzer. Alleles were manually inspected with Peakscanner software (Applied Biosystems), and final allele sizes were determined using TANDEM v.1.08 (Matschiner and Salzburger 2009).

### 2.3 Statistical Analysis

The number of alleles, expected and observed heterozygosity, and further polymorphic information content (PIC), paternity exclusion index (QC), and the genetic identity index (IC) values were calculated using CERVUS v.3.0.7 (Kalinowski et al. 2007) and can be found in Table 1. We used the program GERUD 2.0 (Jones 2005) for parental reconstruction of each clutch, as neonates could be grouped a priori as full or half‐sibs. We used the allele frequency data provided from all samples at the three populations in French Guiana to increase reliability of the parental reconstruction analysis in GERUD 2.0. The program constructs potential mother genotypes, followed by an exhaustive search to determine the minimum number of fathers necessary to explain either the half or full‐sib progeny array when neither parent is known (Jones 2005). For two nest sites, we had potential mother genotypes at hand and included them in the reconstruction, namely, in Kourou Space Center (KSC‐3) and Mont Grand Matoury (MGM‐5). Due to the specific nature of the program, which does not accept missing data, we were unable to include neonates that could not be genotyped at all loci. A summary of the number of individuals used per progeny array is provided in Table 2. To determine if one or two females were involved in the two nests at the same location between years at nature reserve “Les Nouragues – Inselberg”, we pooled clutches by area and determined if only one mother was reconstructed, based on GERUD 2.0. Despite the availability of more and newer programs for reconstructing parental genotypes, we have chosen to utilize GERUD 2.0 because of its conservative approach which has better performance with a reduced amount of data compared to COLONY (Jones and Wang 2010) and CERVUS (Kalinowski et al. 2007). The latter two programs both overestimated the actual number of fathers in a dataset with known relationships (Isberg 2022).

**TABLE 1:** Summary of the eight microsatellite loci amplified in the Smooth‐fronted caiman ( *P. trigonatus* ) in French Guiana with CERVUS 3.0.7 for all available individuals in three populations (*n* = 77).

Locus	Number of alleles	Number of individuals	Ho	He	PIC	QC	IC
**Ptrig02**	**2**	**76**	**0.38**	**0.37**	**0.30**	**0.93**	**0.47**
**Ptrig03**	**6**	**74**	**0.76**	**0.72**	**0.66**	**0.71**	**0.13**
Ptrig04	3	76	0.01	0.04	0.04	0.99	0.96
Ptrig05	2	66	0.05	0.05	0.04	0.99	0.91
**Ptrig06**	**6**	**75**	**0.68**	**0.61**	**0.55**	**0.80**	**0.21**
**Ptrig07**	**4**	**76**	**0.82**	**0.72**	**0.66**	**0.71**	**0.13**
Ptrig08	1	76	0	0	0	1	1
**Ptrig09**	**3**	**72**	**0.31**	**0.38**	**0.31**	**0.93**	**0.45**
All loci	3.4	77	—	0.36	0.32	0.35	< 0.01
**Loci parental reconstruction**	**4.2**	**77**	—	**0.56**	**0.50**	**0.35**	**< 0.01**

*Note:* Given are locus names according to Muniz et al. (2019), number of genotyped individuals, number of alleles found in the population, observed heterozygosity (Ho), expected heterozygosity (He), polymorphic information content (PIC), parental exclusion index (QC), and genetic identity index (IC). The five loci used for parental reconstruction are indicated in bold, and the summary is given at the end of the table (mean number of alleles per locus).

**TABLE 2:** Summary of location, sampling year, clutch ID, number of neonates sampled, number of neonates used in the progeny array, availability of mother genotypes for the specific nest, and the estimated minimum number of fathers.

Location	Year	Clutch ID	Number of neonates sampled	Number of neonates in progeny array	Potential mother genotype available	Minimum number of fathers
Nature Reserve Les Nouragues Inselberg	2017	NOU‐1	15	11	No	2
	2019	NOU‐4	7	5	No	2
Nature Reserve Mont Grand Matoury	2018	MGM‐2	7	5	No	1
	2019	MGM‐5	9	9	Yes	1
Kourou, Space Center	2018	KSC‐3	7	5	Yes	2

## 3 Results

A total of 77 *P. trigonatus* individuals (59 juveniles, 9 subadults, and 9 adults) from the three locations (Figure 2) were genotyped using eight microsatellite loci (Table 1). The overall genetic diversity was very low, with an expected heterozygosity of 0.36 for the eight loci combined. While one locus was monomorphic (Ptrig08), two loci had very low observed heterozygosity and, therefore, a very low polymorphic information content (PIC; Ptrig04, Ptrig05). These three loci were excluded from further analysis, and the remaining five markers were used for parental reconstruction of neonates from five nest locations. While removing the three loci increased the overall PIC to 0.5, the parental exclusion index remained low, at 0.35.

Using GERUD 2.0., we detected multiple paternity in three of the five clutches (60%), where the program identified a minimum of two different fathers (Table 2). The two clutches with a minimum of one father were found in Mont Grand Matoury at two different nest sites. The female sampled in close proximity to MGM‐5 fitted with the genotypes of the progeny and was the mother of that clutch. The second clutch originated from a different female, as the genotype did not fit the progeny genotypes from clutch MGM‐2. The two clutches found at the same nest location in “Les Nouragues – Inselberg” nature reserve in 2017 (NOU‐1) and 2019 (NOU‐4) were not assigned to one female genotype, and both had a minimum of two fathers when analyzed separately. The combined analysis of both clutches gave at least three fathers, indicating that one of the two males might have sired both. However, we lack the statistical power to be certain.

The genotypes of five neonates used for the progeny array in Kourou with five loci did not completely match the genotype of the potential mother sampled close to the nest (KSC‐3). We reduced the loci number to three to include the other two neonates sampled around the nest site (7 neonates, 3 loci). GERUD 2.0 gave two potential mother genotypes for this reduced dataset, indicating two mothers having their nests nearby or sharing the nest site. Nevertheless, the program also identified a minimum of two fathers to the progeny.

## 4 Discussion

This study presents the first evidence that multiple paternity is a common phenomenon in the Smooth‐fronted caiman population of French Guiana. Even though all New World crocodylians exhibit multiple paternity, which seems to be an ancestral state within the group (Isberg 2021; Muniz et al. 2019), we anticipated a lower incidence of multiple paternity due to the sedentary and territorial behavior of the species (Magnusson and Lima 1991; Marioni et al. 2022). The multiple paternity rate was found to be 60%, comparable to that observed in other crocodylian species (Isberg 2022). Multiple mating was observed in the nature reserve “Les Nouragues ‐ Inselberg” and Kourou Space Center, but not in Mont Grand Matoury nature reserve (Figure 2). Such variability might be attributed to population size or density variations, potentially influenced by human activities. Despite monitoring these populations since 2016, reliable population estimates remain elusive due to the infrequency of detection and recapture events. The Mont Grand Matoury population is constrained by limited available habitat, given its proximity to human settlements. Although there is no definitive evidence, there have been reports of caimans being hunted in Mont Grand Matoury for food resources (personal communication to JL). In Brazil, it was shown that subsistence hunting was higher in areas closer to settlements and that dwarf caimans were preferred bushmeat (Muniz et al. 2021). This could potentially lead to a reduction in population density, detectability, and female–male encounters. The Mont Grand Matoury area is more accessible to humans than the Kourou Space Center or the Nouragues Reserve. The Space Center is less accessible to the public, but humans are frequently present, which may result in habituation towards humans and thus a higher detection probability, and less secretive behaviors. The most remote area is the Nouragues Reserve, where caimans could be abundant but are difficult to detect (Lemaire et al. 2018).

Different females used the nest site sampled in Nouragues Reserve in consecutive years. This indicates that nest sites could be a limiting resource due to the requirements of *P. trigonatus* nests being close to small streams, under closed canopy with direct sunlight for only a couple of hours per day, and close/next to termite mounds (Figure 3; Magnusson et al. 1985; Magnusson et al. 1990). The implications of repetitive usage are manifold. Females do not reproduce every year, a pattern commonly observed in crocodylians and other reptiles (Thorbjarnarson 1996), and the interval of reproduction is expected to be 3 years in *P. trigonatus* (Magnusson and Lima 1991). There could be cooperation between females by sharing the nest sites in different years or breeding close to each other. Additionally, females could compete for nest sites as a limiting resource, but such observations have not been made. The same applies to the nest site in Kourou Space Center, as the neonates did not originate from the same mother, and two females should have used nest sites close to each other or shared the same nest site. We found remains of 11 eggs at the nest site but only seven neonates. This is a possible scenario as females have been shown to have overlapping home ranges and seem less territorial (Magnusson and Lima 1991), although we do not know if those observations have been made during the reproductive season.

The adaptive nature of multiple paternity is still discussed due to the difficulties in estimating the costs and benefits for females that mate with more than one male (Isberg 2021; Uller and Olsson 2008). Direct benefits of multiple paternity for the female can be defined as benefits arising from male contributions to parental care or provisioning of resources, both not applicable to crocodylians, as males, in most cases, do not provide parental care or resources (Lang 1987). In this study, we found that nesting sites might be a limiting resource for this highly specialized species and could be a valuable resource that increases mating probabilities for males who could provide those resources in their territory. While larger males could benefit in mating attempts, they do not necessarily have larger home ranges and, therefore, more resources. Specifically in *P. trigonatus*, home range sizes are relatively small and do not correlate with male body size (Marioni et al. 2022). There is still very scarce information on the spatial ecology of this species, and no information is available on habitat choices by males or females.

Additionally, females could be prone to an increased risk of injuries from mating, as is known from other reptile species (Uller and Olsson 2008). However, in cases of low encounter rates with mates, females may benefit from increased sperm availability due to multiple mating. Research indicates that multiple mating can enhance fertilization success through sperm storage, sperm competition, and also give rise to cryptic female choice (Eberhard 2009). However, there is nothing known so far for *P. trigonatus* , as mating was never observed in smooth‐fronted caimans, and no behavioral or experimental studies exist.

Indirect benefits of multiple paternity are increased genetic diversity among the offspring, which could lead to higher plasticity and adaptability during environmental changes, increasing the survival chances and thus the overall fitness of the female (Jennions and Petrie 2000; Taylor et al. 2014). Indirect effects of multiple paternity could also be seen as the consequences at the population level, for example, to maintain genetic diversity in isolated local populations (Isberg 2021; Muniz et al. 2011; Wang et al. 2017), improving the fitness of the offspring related to genetic diversification at the population level (Wang et al. 2017) and increasing effective population size (Sugg and Chesser 1994). However, this mechanism can only work if genetic diversity is not diminished through recent bottlenecks (Isberg 2021). The Guiana lineage of *P. trigonatus* , which includes French Guiana, shows low genetic diversity, possibly due to its divergence from the Amazon lineages 7.5 Ma ago (Bittencourt et al. 2019). However, the benefits of multiple paternity in terms of improved offspring performance have not yet been demonstrated in crocodylians. For example, Zajdel et al. (2019) did not detect any impact of multiple paternity on mass, length, and body condition in offspring of American alligators ( *Alligator mississippiensis* ), suggesting that there is no clear evidence of short‐term fitness benefits for the hatchlings. Additionally, they detected a lower fertility rate in multiple sired clutches that could exert a cost on the female's fitness. Moreover, Lewis et al. (2013) found no impact of multiple paternity on hatching success in Estuarine Crocodile ( *Crocodylus porosus* ). Therefore, we do not think that multiple paternity is a strategy to increase genetic diversity, but rather an adaptation towards low encounter rates based on environmental and social circumstances.

The environmental potential for polygamy (e.g., spatial and temporal availability of territories and food) and social relationships (e.g., population density) influence the probability of multiple paternity (Emlen and Oring 1977). The probability of multiple paternity will increase when the adult populations are male biased (Pipoly et al. 2023). This bias might increase encounter rates between females and males, and sexual coercion by dominant males, which is common in reptiles (Uller and Olsson 2008). Although the adult sex ratio is predicted to be female‐biased in *P. trigonatus* due to temperature‐dependent sex determination (Magnusson et al. 1990), it cannot be confirmed from our long‐term field sampling, as we catch mostly males (2:1, unpublished data). This might not necessarily reflect the accurate adult sex ratio, as males seem to be bolder than females and therefore might be easier to catch. While handling the neonates, we noticed one parent, presumably female, observing us. However, the animal did not leave its refuge or defend the neonates, making it impossible to catch and sample them. If females stay and protect their nests and hatchlings, they probably won't be available for more matings. This could lead to a male‐biased operational sex ratio, meaning there'll be fewer females available for mating in a specific season. We have a low recapture rate in general, which illustrates the cryptic behavior of the species and demonstrates the challenges associated with natural history and life history studies in this species, which was also documented by Magnusson and Lima (1991). However, in scenarios where encounter rates are low, a potential consequence is that females may mate with all males they encounter, to minimize the risk of sperm depletion (Uller and Olsson 2008).

The overall low genetic diversity in *P. trigonatus* in our populations might also explain the low diversity of three microsatellites with our samples that were developed mainly from the Amazon lineage (Muniz et al. 2019). The three markers did not add much value to our analysis due to the low parentage exclusion index and low heterozygosity. Nevertheless, we are confident that our analysis reflects the accurate estimate of multiple paternity in these populations of *P. trigonatus* as GERUD 2.0 is a reliable and very conservative approach for parental reconstruction and has been shown to perform best if marker quality is low (Isberg 2022). However, GERUD 2.0 does not account for genotyping errors. For future population genomics or parental reconstruction, we recommend testing either more microsatellites on local populations or using ddRAD (or other next‐generation sequencing approaches) to increase marker availability and, therefore, resolution.

## 5 Conclusion

We provide the first evidence for multiple paternity in French Guiana's smooth‐fronted caiman, *Paleosuchus trigonatus* . Despite classifying *P. trigonatus* as a least concern species, the low genetic diversity observed in French Guiana populations may indicate an increased vulnerability to environmental changes, especially since *P. trigonatus* might be affected by mercury contamination due to small‐scale artisanal gold mining (Lemaire et al. 2021b) and associated subsistence poaching (Muniz et al. 2021). Although we provide evidence of multiple paternity in our study, this might not be sufficient to maintain genetic diversity, necessitating the country's monitoring of populations to detect trends and planning conservation interventions accordingly, as highlighted in a recent study by Rodriguez‐Cordero et al. (2024).

## Author Contributions

**Carolin Dittrich:** conceptualization (equal), formal analysis (lead), writing – original draft (lead), writing – review and editing (equal). **Rosanna Mangione:** conceptualization (equal), investigation (equal), writing – original draft (equal), writing – review and editing (equal). **Olivier Marquis:** conceptualization (equal), funding acquisition (equal), investigation (equal), writing – review and editing (equal). **Eva Ringler:** conceptualization (equal), methodology (supporting), writing – review and editing (equal). **Jérémy Lemaire:** conceptualization (equal), funding acquisition (lead), investigation (equal), methodology (equal), writing – original draft (equal), writing – review and editing (equal).

## Ethics Statement

Capture of animals and sample collection were performed under permit from the French authorities (Direction Régionale des Territoires et de la Mer) after evaluation by the CSRPN, the regional scientific committee (Permits: R03‐2016‐06‐21‐010, R03‐2017‐11‐17‐006, R03‐2019‐01‐09‐001, and R03‐2019‐10‐24‐007).

## Conflicts of Interest

The authors declare no conflicts of interest.

## Data Availability

The details for the microsatellites (primers, sequences, etc.) and the data on the eight microsatellites for all 77 individuals, together with individual location, sex, status and nest association are provided as a csv‐files and a ReadMe txt file. We supply the data for reviewers in the submission system and will upload it to the data repository of the University of Vienna before publication (https://doi.org/10.5061/dryad.2fqz6131b).

## References

[ece371337-bib-0001] Bittencourt, P. S. , Z. Campos , F. d L. Muniz , et al. 2019. “Evidence of Cryptic Lineages Within a Small South American Crocodilian: The Schneider's Dwarf Caiman *Paleosuchus Trigonatus* (Alligatoridae: Caimaninae).” PeerJ 7: e6580. 10.7717/peerj.6580. 30931177 PMC6433001

[ece371337-bib-0002] Campos, Z. , W. E. Magnusson , and F. Muniz . 2019. “ *Paleosuchus trigonatus* The IUCN Red List of Threatened Species 2019.”: e.T46588A3010035. 10.2305/IUCN.UK.2019-1.RLTS.T46588A3010035.en.

[ece371337-bib-0003] Campos, Z. , F. L. Muniz , A. L. J. Desbiez , and W. E. Magnusson . 2016. “Predation on Eggs of Schneider's Dwarf Caiman, *Paleosuchus trigonatus* (Schneider, 1807), by Armadillos and Other Predators.” Journal of Natural History 50, no. 25–26: 1543–1548. 10.1080/00222933.2016.1155782.

[ece371337-bib-0004] Campos, Z. , T. Sanaiotti , V. Marques , and W. E. Magnusson . 2015. “Geographic Variation in Clutch Size and Reproductive Season of the Dwarf Caiman, *Paleosuchus Palpebrosus*, in Brazil.” Journal of Herpetology 49, no. 1: 95–98. 10.1670/11-224.

[ece371337-bib-0005] Correia, H. E. , A. Abebe , and F. S. Dobson . 2021. “Multiple Paternity and the Number of Offspring: A Model Reveals Two Major Groups of Species.” BioEssays 43, no. 4: 2000247. 10.1002/bies.202000247. 33491804

[ece371337-bib-0006] Dobson, F. S. , H. E. Correia , and A. Abebe . 2024. “How Much Multiple Paternity Should We Expect? A Study of Birds and Contrast With Mammals.” Ecology and Evolution 14, no. 3: e11054. 10.1002/ece3.11054. 38435004 PMC10905237

[ece371337-bib-0007] Eberhard, W. G. 2009. “Postcopulatory Sexual Selection: Darwin's Omission and Its Consequences.” Proceedings of the National Academy of Sciences 106: 10025–10032. 10.1073/pnas.0901217106. PMC2702800 19528642

[ece371337-bib-0008] Emlen, S. T. , and L. W. Oring . 1977. “Ecology, Sexual Selection, and the Evolution of Mating Systems.” Science 197, no. 4300: 215–223. 10.1126/science.327542. 327542

[ece371337-bib-0009] Griffith, S. C. , I. P. F. Owens , and K. A. Thuman . 2002. “Extra Pair Paternity in Birds: A Review of Interspecific Variation and Adaptive Function.” Molecular Ecology 11, no. 11: 2195–2212. 10.1046/j.1365-294X.2002.01613.x. 12406233

[ece371337-bib-0010] Isberg, S. R. 2021. “Crocodilians Are Promiscuous but Not to the Benefit of Heterozygosity.” In Conservation Genetics of New World Crocodilians, edited by R. B. Zucoloto , P. S. Amavet , L. M. Verdale , and I. P. Farias , 153–170. Springer. 10.1007/978-3-030-56383-7_6.

[ece371337-bib-0011] Isberg, S. R. 2022. “How Many Fathers? Study Design Implications When Inferring Multiple Paternity in Crocodilians.” Ecology and Evolution 12: e9379. 10.1002/ece3.9379. 36225824 PMC9534745

[ece371337-bib-0013] Jennions, M. D. , and M. Petrie . 2000. “Why Do Females Mate Multiply? A Review of the Genetic Benefits.” Biological Reviews 75, no. 1: 21–64. 10.1111/j.1469-185X.1999.tb00040.x. 10740892

[ece371337-bib-0014] Jones, A. G. 2005. “GERUD 2.0: A Computer Program for the Reconstruction of Parental Genotypes From Half‐Sib Progeny Arrays With Known or Unknown Parents.” Molecular Ecology Notes 5, no. 3: 708–711. 10.1111/j.1471-8286.2005.01029.x.

[ece371337-bib-0015] Jones, O. R. , and J. Wang . 2010. “COLONY: A Program for Parentage and Sibship Inference From Multilocus Genotype Data.” Molecular Ecology Resources 10, no. 3: 551–555. 10.1111/j.1755-0998.2009.02787.x. 21565056

[ece371337-bib-0016] Kalinowski, S. T. , M. L. Taper , and T. C. Marshall . 2007. “Revising How the Computer Program CERVUS Accommodates Genotyping Error Increases Success in Paternity Assignment.” Molecular Ecology 16, no. 5: 1099–1106. 10.1111/j.1365-294X.2007.03089.x. 17305863

[ece371337-bib-0017] Kokko, H. , H. Klug , and M. D. Jennions . 2014. “Mating Systems.” In The Evolution of Insect Mating Systems, edited by D. M. Shuker and L. W. Simmons , 42–58. Oxford University press.

[ece371337-bib-0018] Lang, J. W. 1987. “Crocodilian Behaviour: Implications for Management.” In Wildlife Management: Crocodiles and Alligators, edited by G. J. W. Webb , S. C. Manolis , and P. J. Whitehead , 273–294. Surrey Beatty.

[ece371337-bib-0019] Lemaire, J. , F. Brischoux , O. Marquis , R. Mangione , and P. Bustamante . 2021a. “Variation of Total Mercury Concentrations in Different Tissues of Three Neotropical Caimans: Implications for Minimally Invasive Biomonitoring.” Archives of Environmental Contamination and Toxicology 81: 15–24. 10.1007/s00244-021-00846-y. 33899129

[ece371337-bib-0020] Lemaire, J. , O. Marquis , P. Bustamante , R. Mangione , and F. Brischoux . 2021b. “I Got It From My Mother: Inter‐Nest Variation of Mercury Concentration in Neonate Smooth‐Fronted Caiman ( *Paleosuchus trigonatus* ) Suggests Maternal Transfer and Possible Phenotypical Effects.” Environmental Research 194: 110494. 10.1016/j.envres.2020.110494. 33220243

[ece371337-bib-0021] Lemaire, J. , O. Marquis , D. Oudjani , and P. Gaucher . 2018. “Habitat Use and Behaviour of Schneider's Dwarf Caiman (*Paleosuchus Trigonatus*, Schneider 1801) in Nouragues Reserve. French Guiana.” Crocodile Specialist Group Newsletter 37, no. 2: 18–21. https://hal.science/hal‐02409648v1.

[ece371337-bib-0022] Lewis, J. L. , N. N. FitzSimmons , M. L. Jamerlan , J. C. Buchan , and G. C. Grigg . 2013. “Mating Systems and Multiple Paternity in the Estuarine Crocodile ( *Crocodylus porosus* ).” Journal of Herpetology 47, no. 1: 24–33. 10.1670/10-303.

[ece371337-bib-0023] Magnusson, W. E. , E. V. da Silva , and A. P. Lima . 1987. “Diets of Amazonian Crocodilians.” Journal of Herpetology 21, no. 2: 85–95. 10.2307/1564468.

[ece371337-bib-0024] Magnusson, W. E. , and A. P. Lima . 1991. “The Ecology of a Cryptic Predator, *Paleosuchus Trigonatus*, in a Tropical Rainforest.” Journal of Herpetology 25, no. 1: 41–48. 10.2307/1564793.

[ece371337-bib-0025] Magnusson, W. E. , A. P. Lima , J.‐M. Hero , T. M. Sanaiotti , and M. Yamakoshi . 1990. “ *Paleosuchus Trigonatus* Nests: Sources of Heat and Embryo Sex Ratios.” Journal of Herpetology 24, no. 4: 397–400. 10.2307/1565056.

[ece371337-bib-0026] Magnusson, W. E. , A. P. Lima , and R. M. Sampaio . 1985. “Sources of Heat for Nests of Paleosuchus Trigonatus and a Review of Crocodilian Nest Temperatures.” Journal of Herpetology 19, no. 2: 199–207. 10.2307/1564173.

[ece371337-bib-0027] Marioni, B. , W. E. Magnusson , R. C. Vogt , and F. Villamarín . 2022. “Home Range and Movement Patterns of Male Dwarf Caimans (Paleosuchus Palpebrosus and *Paleosuchus Trigonatus*) Living in Sympatry in Amazonian Floodplain Streams.” Neotropical Biodiversity 8, no. 1: 156–166. 10.1080/23766808.2022.2061292.

[ece371337-bib-0028] Matschiner, M. , and W. Salzburger . 2009. “TANDEM: Integrating Automated Allele Binning Into Genetics and Genomics Workflows.” Bioinformatics 25: 1982–1983. 10.1093/bioinformatics/btp303. 19420055

[ece371337-bib-0029] Muniz, F. L. , R. Da Silveira , Z. Campos , W. E. Magnusson , T. Hrbek , and I. P. Farias . 2011. “Multiple Paternity in the Black Caiman (*Melanosuchus Niger*) Population in the Anavilhanas National Park, Brazilian Amazonia.” Amphibia‐Reptilia 32, no. 3: 428–434. 10.1163/017353711X587741.

[ece371337-bib-0030] Muniz, F. L. , A. M. Ximenes , P. S. Bittencourt , et al. 2019. “Detecting Population Structure of *Paleosuchus trigonatus* (Alligatoridae: Caimaninae) Through Microsatellites Markers Developed by Next Generation Sequencing.” Molecular Biology Reports 46, no. 2: 2473–2484. 10.1007/s11033-019-04709-7. 30852718

[ece371337-bib-0031] Muniz, F., L , Z. Campos , P. Bittencourt S , T. Hrbek , and I. Farias P . 2021. “Report of Poaching of Dwarf Caimans *Paleosuchus* spp. (Alligatoridae: Caimaninae) for Meat Consumption in Northern Brazilian Amazon.” Herpetology Notes 14: 661–665.

[ece371337-bib-0032] Parker, G. A. 1970. “Sperm Competition and Its Evolutionary Consequences in the Insects.” Biological Reviews 45, no. 4: 525–567. 10.1111/j.1469-185X.1970.tb01176.x.

[ece371337-bib-0033] Parker, G. A. 2006. “Sexual Conflict Over Mating and Fertilization: An Overview.” Philosophical Transactions of the Royal Society, B: Biological Sciences 361, no. 1466: 235–259. 10.1098/rstb.2005.1785. PMC1569603 16612884

[ece371337-bib-0034] Pipoly, I. , R. Duffy , G. Mészáros , et al. 2023. “Multiple Paternity Is Related to Adult Sex Ratio and Sex Determination System in Reptiles.” Journal of Evolutionary Biology 36, no. 6: 935–944. 10.1111/jeb.14185. 37259484

[ece371337-bib-0035] Pizzari, T. , and N. Wedell . 2013. “The Polyandry Revolution.” Philosophical Transactions of the Royal Society, B: Biological Sciences 368, no. 1613: 20120041. 10.1098/rstb.2012.0041. PMC3576576 23339233

[ece371337-bib-0036] Rodriguez‐Cordero, A. L. , S. A. Balaguera‐Reina , B. A. Gross , M. Munn , and L. D. Densmore III . 2024. “Assessing Abundance–Suitability Models to Prioritize Conservation Areas for the Dwarf Caimans in South America.” Ecology and Evolution 14, no. 9: e70235. 10.1002/ece3.70235. 39219570 PMC11362219

[ece371337-bib-0037] Sambrook, J. , E. F. Fritsch , and T. Maniatis . 1989. Molecular Cloning: A Laboratory Manual. 2nd ed. Cold Spring Harbor Laboratory Press.

[ece371337-bib-0038] Sugg, D. W. , and R. K. Chesser . 1994. “Effective Population Sizes With Multiple Paternity.” Genetics 137, no. 4: 1147–1155. 10.1093/genetics/137.4.1147. 7982568 PMC1206061

[ece371337-bib-0039] Taylor, M. L. , T. A. Price , and N. Wedell . 2014. “Polyandry in Nature: A Global Analysis.” Trends in Ecology & Evolution 29, no. 7: 376–383. 10.1016/j.tree.2014.04.005. 24831458

[ece371337-bib-0040] Thorbjarnarson, J. B. 1996. “Reproductive Characteristics of the Order Crocodylia.” Herpetologica 52, no. 1: 8–24. https://www.jstor.org/stable/3892951.

[ece371337-bib-0041] Uller, T. , and M. Olsson . 2008. “Multiple Paternity in Reptiles: Patterns and Processes.” Molecular Ecology 17, no. 11: 2566–2580. 10.1111/j.1365-294X.2008.03772.x. 18452517

[ece371337-bib-0042] Wang, H. , P. Yan , S. Zhang , et al. 2017. “Multiple Paternity: A Compensation Mechanism of the Chinese Alligator for Inbreeding.” Animal Reproduction Science 187: 124–132. 10.1016/j.anireprosci.2017.10.016. 29103625

[ece371337-bib-0043] West‐Eberhard, M. J. 1983. “Sexual Selection, Social Competition, and Speciation.” Quarterly Review of Biology 58, no. 2: 155–183. 10.1086/413215.

[ece371337-bib-0044] Zajdel, J. , S. L. Lance , T. R. Rainwater , P. M. Wilkinson , M. D. Hale , and B. B. Parrott . 2019. “Mating Dynamics and Multiple Paternity in a Long‐Lived Vertebrate.” Ecology and Evolution 9, no. 18: 10109–10121. 10.1002/ece3.5438. 31632641 PMC6787947

